# Defining Priorities for Future Research: Results of the UK Kidney Transplant Priority Setting Partnership

**DOI:** 10.1371/journal.pone.0162136

**Published:** 2016-10-24

**Authors:** Simon R. Knight, Leanne Metcalfe, Katriona O’Donoghue, Simon T. Ball, Angela Beale, William Beale, Rachel Hilton, Keith Hodkinson, Graham W. Lipkin, Fiona Loud, Lorna P. Marson, Peter J. Morris

**Affiliations:** 1 Centre for Evidence in Transplantation, Clinical Effectiveness Unit, Royal College of Surgeons of England, London, United Kingdom; 2 Nuffield Department of Surgical Sciences, University of Oxford, Oxford, United Kingdom; 3 James Lind Alliance, National Institute for Health Research Evaluation, Trials and Studies Coordinating Centre, University of Southampton, Southampton, United Kingdom; 4 University Hospitals Birmingham NHS Foundation Trust, Birmingham, United Kingdom; 5 British Renal Society, Lichfield, United Kingdom; 6 National Kidney Federation, Worksop, United Kingdom; 7 Guy’s and St. Thomas’ NHS Foundation Trust, London, United Kingdom; 8 British Transplantation Society, Macclesfield, United Kingdom; 9 Kidney Research UK, Peterborough, United Kingdom; 10 Renal Association, Petersfield, United Kingdom; 11 British Kidney Patient Association, Alton, United Kingdom; 12 University of Edinburgh, Edinburgh, United Kingdom; Universidade de Sao Paulo, BRAZIL

## Abstract

**Background:**

It has been suggested that the research priorities of those funding and performing research in transplantation may differ from those of end service users such as patients, carers and healthcare professionals involved in day-to-day care. The Kidney Transplant Priority Setting Partnership (PSP) was established with the aim of involving all stakeholders in prioritising future research in the field.

**Methods:**

The PSP methodology is as outlined by the James Lind Alliance. An initial survey collected unanswered research questions from patients, carers and clinicians. Duplicate and out-of-scope topics were excluded and the existing literature searched to identify topics answered by current evidence. An interim prioritisation survey asked patients and professionals to score the importance of the remaining questions to create a ranked long-list. These were considered at a final consensus workshop using a modified nominal group technique to agree a final top ten.

**Results:**

The initial survey identified 497 questions from 183 respondents, covering all aspects of transplantation from assessment through to long-term follow-up. These were grouped into 90 unanswered “indicative” questions. The interim prioritisation survey received 256 responses (34.8% patients/carers, 10.9% donors and 54.3% professionals), resulting in a ranked list of 25 questions that were considered during the final workshop. Participants agreed a top ten priorities for future research that included optimisation of immunosuppression (improved monitoring, choice of regimen, personalisation), prevention of sensitisation and transplanting the sensitised patient, management of antibody-mediated rejection, long-term risks to live donors, methods of organ preservation, induction of tolerance and bioengineering of organs. There was evidence that patient and carer involvement had a significant impact on shaping the final priorities.

**Conclusions:**

The final list of priorities relates to all stages of the transplant process, including access to transplantation, living donation, organ preservation, post-transplant care and management of the failing transplant. This list of priorities will provide an invaluable resource for researchers and funders to direct future activity.

## Introduction

Kidney transplantation is arguably the most successful treatment for end-stage kidney failure in suitable patients, with evidence that demonstrates cost effectiveness, improved survival and improved quality of life in comparison with patients remaining on dialysis [1,2]. Over 3,000 kidney transplants are performed each year in the United Kingdom, with 5-year graft survival of around 85% [3].

Despite the success of kidney transplantation there are still challenges. The limited donor pool means that access to transplantation is not universal, with a median waiting time of 1,022 days for adult patients in the United Kingdom resulting in a waiting list of around 5,600 patients at any one time [3]. Furthermore, despite successes in improving short-term graft survival, effective strategies to reduce longer-term graft loss have proved elusive [3,4].

Given these challenges, it is perhaps not surprising that the field of kidney transplantation has a very active research community. The research agenda has typically been set by individual researchers or industry, with over one third of randomised controlled trials in transplantation receiving industry funding [5]. It has been suggested in other areas of health research that the research priorities of these groups may differ from those of end service users such as patients, carers and healthcare professionals involved in day-to-day care [6,7]. Whilst there are no published studies examining the differences in research priorities between transplant patients and professionals, two studies have reported differences between groups in patients with chronic kidney disease (CKD), the scope of which included transplantation [8,9]. In particular, patients tended to prioritise research into difficult to treat symptoms and side effects such as fatigue and restless legs more highly than professionals.

Increasing recognition of a potential mismatch between the priorities of end service users and researchers/funders has led to a drive towards involving healthcare professionals, patients and carers in projects identifying and prioritising topics for research. This approach has been championed by the James Lind Alliance in the UK, a not-for-profit organisation now a part of the National Institute for Health Research (NIHR) Evaluation, Trials and Studies Coordinating Centre (NETSCC). These “Priority Setting Partnerships” (PSPs) have been successful in a number of areas of medicine, and have led to successful funding of many of the research questions identified [10–14].

The aims of the present study were (i) to identify unanswered research questions in the field of kidney transplantation from end service users (patients, carers and healthcare professionals) and (ii) to prioritise these questions according to the needs of these groups, for use in future decision making by funders and researchers.

## Methods

The Kidney Transplant Priority Setting Partnership followed the methodology described in detail in the James Lind Alliance (JLA) guidebook [15]. In keeping with guidance from the JLA, priority setting partnerships do not require specific ethical approval as they are considered by the NHS Research Ethics Service (NRES) as service evaluation and development projects. The ethical guidelines set out in the JLA guidebook were followed throughout the process. Participation in the surveys and workshop was entirely voluntary, and specific written consent (via the online survey if used) for publication of research questions submitted/prioritised was sought. Participants in the final workshop signed a declaration in advance to state that they agreed with the principles of the project and to identify any potential conflicts of interest. The methods used are included here in outline form. The PSP took place between January 2014 and February 2016.

### Organisation and scope

National patient and professional organisations and charities involved in kidney transplantation were contacted about the project and invited to contribute to a steering group. This group included representation from the Centre for Evidence in Transplantation (CET), British Transplantation Society (BTS), Renal Association (RA), British Renal Society (BRS), Kidney Research UK (KRUK), the National Kidney Federation (NKF) and the British Kidney Patient Association (BKPA). The group was chaired by an experienced advisor from the JLA (LM). The steering group developed the protocol for the project and was involved in all stages of its management. It included transplant surgeons, nephrologists, transplant recipients, living donors and carers. Additional partner organisations were invited to take part in the process by involving their members in the surveys and helping to promote the process. A full list of partner organisations is included in the Supporting Information (S1 File).

The steering group also defined the scope of the project. This incorporated all stages of the transplant process, including access to the waiting list and pre-transplant assessment, the transplant procedure itself, post-transplant care in the short and long-term, management of the failed transplant and issues surrounding retransplantation and living-donor transplantation. Scope included both adult and paediatric patients and their carers and clinicians. In order to maintain the focus of the process, it excluded management of end-stage kidney failure other than transplantation, donor selection and management (other than living donors) and issues specific to combined organ transplants.

### Identification of potential research questions

In order to identify potential research questions, an online survey was created to collect possible questions, along with the demographic details of the respondents. The survey was open-ended, simply asking “What unanswered questions about kidney transplantation and living donation would you like to see answered by research?” with space for up to three questions to be submitted. The survey was piloted by members of partner organisations and the wording refined according to feedback. It was also made available in paper format to improve access. The paper version included the same instructions and questions with the same wording as the online version, and was made available on request. The survey was open to responses between October and December 2014, and promoted via the steering group, partner organisations and other interested individuals. Methods of dissemination included websites, blogs, social media, society/organisation e-mailings and print newsletters distribution of flyers at conferences and events and display of posters and leaflets in transplant centres. The survey was open to all transplant recipients and those on the transplant waiting list, their carers, live kidney donors and professionals involved in the care of kidney transplant recipients in the UK. As the purpose was to collect as many potential research questions as possible, multiple submissions from the same respondent were allowed.

The steering group also identified two recent previous surveys from the BTS and KRUK to which respondents were asked to submit potential research questions. Questions from these surveys that fell within the scope of the PSP were also included, although demographic information were not available.

### Refinement of questions and identification of existing literature

The raw submissions were imported into a custom designed MySQL database. Responses were reviewed by two researchers and those that were out of scope (as defined above), or that could not be translated into an answerable research question, were identified. These questions were reviewed by the entire steering group and only excluded if all members agreed.

The remaining questions were grouped into similar themes using a pre-defined taxonomy to form a set of “indicative” research questions that covered the scope of all questions submitted. These indicative questions were expressed in the PICO (population, intervention, comparator, outcomes) format as far as possible, although many questions were broad and all four parameters were not defined. Classification and grouping was initially performed by two researchers, with the results discussed in detail by the entire steering group to agree the final indicative questions.

Two experienced systematic reviewers reviewed the transplant literature for each of the remaining indicative uncertainties in order to assess the extent of the existing evidence. The primary sources for this review were the Cochrane Library and the Transplant Library (www.transplantlibrary.com), which includes all randomised controlled trials and systematic reviews (to a minimum quality standard) published in the field of solid organ transplantation between 1970 and present, identified from searches of MEDLINE the Cochrane Library and hand searches of conference proceedings. The purpose of the literature review was to identify any of the questions that were considered adequately answered by the existing literature. In order for a question to be considered answered, an existing up-to-date systematic review with a clear conclusion had to be identified. Questions with no available review, outdated reviews or with an existing review that expressed uncertainty in its conclusions (due to lack of evidence, poor quality evidence or imprecision in effect size) were considered unanswered. Questions considered answered were discussed in detail by the steering group prior to exclusion from the remainder of the process. A full description of the strategy and search terms used to identify existing reviews is provided in the Supporting Information (S2 File).

### Interim prioritisation

In order to make a more manageable prioritisation survey (based upon previous JLA experience), remaining indicative questions submitted by three or more individuals to the initial survey were brought forward for prioritisation. A second online survey was designed and piloted in which respondents were asked to rate each remaining question on a five-point Likert scale from “Not at all important” to “Extremely important”. Following an initial pilot, a glossary of terms was also developed to help non-professional respondents understand some of the medical terminology used in the questions. Demographic characteristics of respondents were also collected, including age, sex, geographical region, ethnicity and role. The second survey was open from October to December 2015, and was promoted in a similar way to the initial survey. All respondents to the initial survey were contacted to participate in prioritisation, although this survey was also open to individuals who had not previously participated in the process. Only one response per participant was allowed.

Responses to the prioritisation survey were collated and the Likert scale coded numerically from 1 (Not at all important) to 5 (Extremely important). The overall mean score as well as the mean score for patients, donors and professionals were calculated for each question. Results were reviewed by the steering group, and the decision was made to take the top 25 ranked questions overall through to the final workshop.

### Final prioritisation workshop

A final one-day workshop was held in London in February 2016. The aim of the workshop was to agree a top-ten list of prioritised questions for future research. Professionals (including nephrologists, transplant surgeons and allied health professionals), patients and donors from partner organisations were invited to attend and contribute via contact details submitted to the two surveys, as well as organisation mailing lists, social media and websites. Participants were selected with the aim of broad representation of all involved groups and organisations. We initially planned to include around 25 participants in the final workshop, based upon optimal numbers for interaction during previous JLA PSPs, as well as budget and space constraints. Due to illness and unexpected work commitments, 20 individuals eventually participated.

The workshop took the form of a modified nominal group process facilitated by experienced JLA advisors. Participants were asked to rank the remaining 25 questions prior to attending the workshop, and in an initial small group session these responses were discussed to identify any agreement in the top and bottom three priorities. In a second small group session the groups were asked to rank the uncertainties based upon this discussion. Groups were balanced to ensure equal professional and non-professional involvement and the facilitators encouraged all participants to take part and voice their opinions. Results of this ranking process from each small group were fed back to the meeting as a whole, with agreement in the top and bottom ranked questions highlighted. A further small group round (with different group composition) took place to revise ranking based upon this feedback. In the final whole group session, the results of the small group discussions were used to suggest a top-ten ranking, and discussion took place as to whether items should be removed or inserted into the top ten. Where there was disagreement, all participants were asked to vote in order to reach a decision. The results of the interim prioritisation survey (including overall rankings and those from patients, donors and professionals) were made available on request throughout the meeting to help inform debate and decision making. At the end of the meeting, a final top-ten questions were agreed as priorities for future research.

## Results

### Identification of research questions

The initial survey received 440 potential research questions from 183 respondents. Responses were from a mixture of professionals (36.1%), patients (37.7%), carers (2.2%) and live donors (21.9%), although some donors also identified themselves as carers for a transplant recipient. Full demographic details of respondents are described in Table 1. 57 further questions were added from previous surveys from KRUK and the BTS, giving a total of 497 potential research questions.

**Table 1:** Demographics of respondents to the initial survey.

Characteristic	N (%)
**Role**	
Patient	69 (37.7)
Waiting list	10 (5.5)
Transplant recipient	59 (32.2)
Carer	4 (2.2)
Live donor	40 (21.9)
Healthcare professional	66 (36.1)
Nephrologist/physician	39 (21.3)
Transplant Surgeon	14 (7.7)
Nurse/co-ordinator	4 (2.2)
Clinical scientist	5 (2.7)
Other	4 (2.2)
Other	4 (2.2)
**Age**	
Less than 18 years	0 (0)
18–24 years	3 (1.6)
25–34 years	17 (9.3)
35–44 years	39 (21.3)
45–54 year	50 (27.3)
55–64 years	38 (20.8)
65–74 years	27 (14.8)
75 or more years	4 (2.2)
Not specified	5 (2.7)
**Sex**	
Male	91 (49.7)
Female	85 (46.4)
Not specified	7 (3.8)
**Ethnicity**	
White	161 (88.0)
Mixed ethnicity	2 (1.1)
Asian/Asian British	12 (6.6)
Black/African/Caribbean/Black British	2 (1.1)
Not Specified	6 (3.3)

Submitted questions covered the breadth of kidney transplantation, and were categorised as relating to recipient assessment, recipient education, access to the waiting list, organ allocation, management of highly sensitised recipients, organ preservation and reconditioning, live donor transplantation, non-directed donation, tissue typing and immunology, ABO and HLA incompatible transplantation, surgical technique and intra-operative management, post-transplant complications, acute and chronic rejection, recurrent disease, psychosocial outcomes, diet and lifestyle, management of the failing transplant, immunosuppression and tolerance, paediatric and adolescent transplantation and organ bioengineering.

### Refinement of questions and identification of existing literature

**Fig 1:**
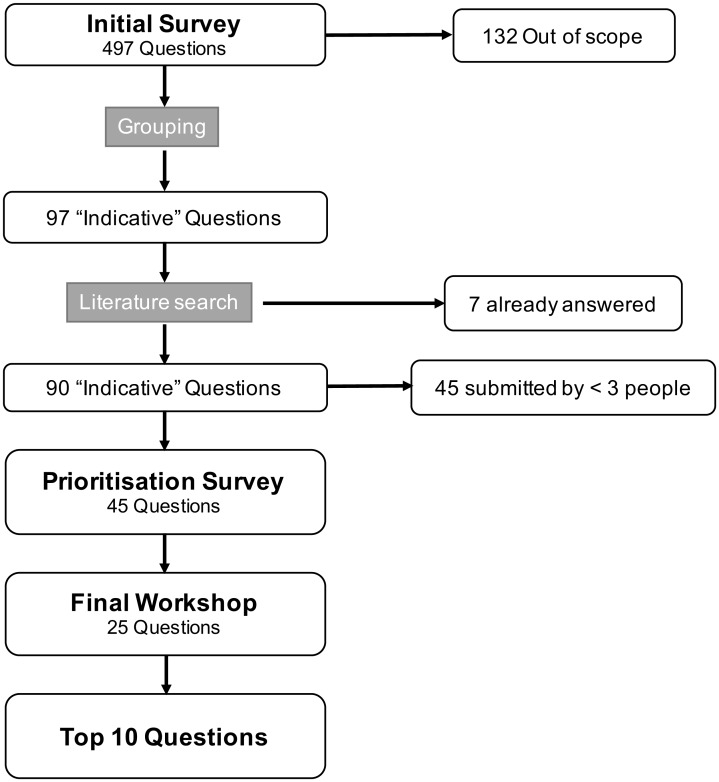
Flow of submitted research questions through the prioritisation process.

The flow of questions through the PSP process is outlined in Fig 1. Of the 497 submitted questions, 132 were out of scope or could not be framed as a research question. The remaining submissions were collated into 97 indicative questions. Seven of these were considered by the steering group to be adequately answered by existing research, and were excluded from the remainder of the process. Of the remaining 90 questions, 45 were submitted by three or more people and were taken forward for prioritisation (S3 File).

### Interim prioritisation

The interim prioritisation survey received 256 responses from a mixture of patients (30.5%), carers (4.3%), donors (10.9%) and professionals (54.3%). Full demographic details of those responding are described in Table 2. Responses were analysed and the top 25 ranked questions overall were taken through to the final workshop (Table 3).

**Table 2:** Demographics of respondents to the prioritisation survey.

Characteristic	N (%)
**Role**	
Patient	78 (30.5)
Waiting list	10 (3.9)
Transplant recipient	68 (26.6)
Carer	11 (4.3)
Live donor	28 (10.9)
Healthcare professional	139 (54.3)
Nephrologist/physician	71 (27.7)
Transplant Surgeon	26 (10.2)
Nurse/co-ordinator	21 (8.2)
Clinical scientist	11 (4.3)
Pharmacist	6 (2.3)
Other	4 (1.6)
**Age**	
Less than 18 years	1 (0.4)
18–24 years	2 (0.8)
25–34 years	19 (7.4)
35–44 years	50 (19.5)
45–54 year	83 (32.4)
55–64 years	63 (24.6)
65–74 years	31 (12.1)
75 or more years	5 (2.0)
Not specified	2 (0.8)
**Sex**	
Male	113 (44.1)
Female	130 (50.8)
Not specified	13 (5.1)
**Ethnicity**	
White	222 (86.7)
Mixed ethnicity	4 (1.6)
Asian/Asian British	10 (3.9)
Black/African/Caribbean/Black British	1 (0.4)
Not Specified	19 (7.4)

**Table 3:** The top 25 ranked questions from the prioritisation survey.

Question	Rank			
	Overall	Patients/carers	Professionals	Donors
Which treatments work best to prolong the life of the kidney transplant (for example different immunosuppression, blood pressure control)?	1	1	1	2
Is there a reliable way for us to assess the suitability of individual organs for transplantation, and to predict outcomes?	2	13	2 (=)	1
What is the best way to treat vascular or antibody mediated rejection?	3	8	2 (=)	12 (=)
How can we prevent sensitisation in patients with a failing transplant, to improve their chances of another successful transplant? (e.g. removal of the transplant, withdrawal of immunosuppressive medicines or continuation of these medicines?)	4	7	6	5
How can immunosuppression be personalised to the individual patient to improve the results of transplantation?	5	4 (=)	5	15 (=)
What is the best combination of immunosuppressive drugs following kidney transplantation (for example azathioprine or mycophenolate, belatacept, generic or proprietary (brand-name) drugs)?	6	2	11	12 (=)
How can we match organs to recipients to ensure the best overall outcomes (for example by age, nephron dosing)?	7	4 (=)	7	6
What tests are required to determine whether a transplant is a suitable option for a patient?	8	6	8	20 (=)
For which patients is transplantation not suitable (considering factors such as age, body mass index, history of cancer, co-morbidities)?	9	24	4	24 (=)
What is the best method of sharing deceased donor kidneys to ensure fair access to all age groups whilst minimising waiting times?	10	11 (=)	17	7
How can we ensure fair and equal access to transplantation across the UK?	11	9	16	15 (=)
Can we improve monitoring of the level of immunosuppression to achieve better balance between risk of rejection and side effects (for example T-cell or B-cell ELISPOT, point-of-care tacrolimus monitoring, MMF monitoring)?	12	17 (=)	12 (=)	22
What techniques to preserve and transport the kidney before transplantation allow increased preservation times and/or improve results (for example machine perfusion, normothermic reconditioning)?	13	3	22	14
What are the long-term health risks to the living kidney donor?	14	22	12 (=)	19
What approaches improve outcomes in adolescent and young adult kidney transplant recipients?	15	17 (=)	14	30
Does routine screening for and treatment of donor-specific antibodies improve outcomes? What is the most effective treatment?	16	25	9	28
How can we increase the number of potential living donors coming forward, and the proportion proceeding to donation?	17	10	23	3
How can we improve transplant rates in highly-sensitised patients?	18	28	10	18
Which combinations of immunosuppressive drugs can minimise side effects in kidney transplant recipients (such as infections, diarrhoea, malignancy)?	19	11 (=)	19	29
How can we encourage tolerance to the transplant to prevent or reduce the need for immunosuppression (for example by use of T-regulatory cells, induction of haemoxygenase 1)?	20	20	20	10
For blood group incompatible transplants, which treatments most effectively reduce antibody levels and improve the safety and outcomes of the operation?	21	21	21	26
Can bioengineered organs be developed to be as safe and effective as human-to-human transplants? How can this be achieved?	22	16	25	9
Can adding substances to the storage or perfusion solution for the kidney improve preservation and overall results (for example oxygenation, EPO, complement inhibitors, stem cells, scavenger molecules)?	23	19	26	24 (=)
What is the best way of educating patients about transplantation before their operation?	24	15	28	20 (=)
How do we prevent the original cause of kidney failure returning (for example glomerulonephritis) following kidney transplant?	25	26	29	4

Respondents were asked to score questions on a five-point Likert scale, and the mean score within each group (patients, professionals and donors) was used to rank questions from highest to lowest score. (=) indicates questions ranked equally.

There was evidence of a difference in priorities between patients, donors and professionals. Of the 25 top-ranked questions, there was a difference in rankings of greater than 5 places between patients and professionals for 16, between donors and professionals for 18 and between patients and donors for 17. Whilst all groups prioritised questions regarding improving long-term transplant outcomes, patients tended to prioritise questions about immunosuppression (including side effects), organ preservation and equity of access. Professionals prioritised questions about assessment of patient and organ suitability and management of antibody mediated rejection. Donors prioritised questions regarding assessment of organ suitability and promotion of living donation. The questions taken to the final workshop represented a mixture of those prioritised by all three groups.

### Final prioritization workshop

Twenty participants took part in the final workshop, representing patients (7), donors (4) and professionals (9). During the day, it was decided that two of the questions had some degree of overlap (regarding organ preservation and conditioning) and the group agreed to merge them together. The top ten research priorities agreed are listed in Table 4. They cover all stages of the transplant process, including optimisation of immunosuppression (improved monitoring, choice of regimen, personalisation), prevention of sensitisation and transplanting the sensitised patient, management of antibody-mediated rejection, long-term risks to live donors, methods of organ preservation, induction of tolerance and bioengineering of organs.

**Table 4:** Kidney Transplant PSP top ten priorities for future research.

Question
What is the best way to treat vascular or antibody-mediated acute rejection?
How can immunosuppression be personalised to the individual patients to improve the results of transplantation?
How can we prevent sensitisation in patients with a failing transplant, to improve their chances of another successful transplant (e.g. removal of the transplant, withdrawal of immunosuppressive medicines or continuation of these medicines?)
Can we improve monitoring of the level of immunosuppression to achieve better balance between risk of rejection and side effects? (e.g. T-cell or B-cell ELISPOT, point-of-care tacrolimus monitoring, MMF monitoring)
How can we improve transplant rates in highly sensitised patients?
What are the long-term health risks to the living kidney donor?
How can we encourage tolerance to the transplant to prevent or reduce the need for immunosuppression? (e.g. by use of T-regulatory cells, induction of haemoxygenase 1)
What is the best combination of immunosuppressive drugs following kidney transplantation? (e.g. azathioprine or mycophenolate, belatacept, generic or proprietary (brand-name) drugs)
What techniques to preserve, condition and transport the kidney before transplantation allow increased preservation times and/or improve results? (e.g. machine perfusion, normothermic reconditioning, addition of agents to the perfusate)
Can bioengineered organs be developed to be as safe as human-to-human transplants? How can this be achieved?

The order of questions does not reflect priority.

Of particular note was the desire of the participants in the final workshop to include realistic questions achievable in the short term (for example those relating to the management of immunosuppression) as well as questions that seem much further away from reality such as the development of bioengineered organs and induction of tolerance. The most highly prioritised question from the interim survey (“What is the best strategy to prolong the life of the transplant kidney?”) was felt by the group to be too generic and covered by many of the more specific questions under consideration, and so was not included in the final top ten.

### Impact of non-professional involvement

A number of questions considered during the process were submitted by non-professionals, and would not have been considered without their involvement. Of the 90 indicative questions 41 (45.5%) were submitted by professionals alone and 14 (15.6%) were submitted by non-professionals alone; the remainder were submitted by both groups. Four questions submitted by patients alone were taken through to the prioritisation stage. Of the final top ten questions, six were originally submitted by both patients and professionals, and four by professionals alone.

The final top ten reflected the mixed priorities of all groups. Of the ten, five appeared in the patient top ten during the prioritisation survey, four in the professional top ten, and two in the donor top ten. Four of the final ten were ranked more than five places higher by patients than professionals, and four were ranked more than five places higher by professionals.

### Dissemination

The final list of priorities, as well as all research questions identified, have been made available on the internet through both the PSP website (www.transplantpsp.org/kidney) and the JLA website (http://www.jla.nihr.ac.uk). They were also disseminated to all partner organisations and participants involved in the process, many of whom are involved in or fund transplant research. The James Lind Alliance is part of the National Institute for Health Research (NIHR), one of the largest funders of health research in the UK. The results of the Kidney Transplant PSP have been discussed with NIHR representatives in detail with the aim of taking forward priorities for future specific funding calls.

## Discussion

The Kidney Transplant PSP brought patients, donors and healthcare professionals together for the first time in the United Kingdom to collect and prioritise topics for future research. The list of research questions generated will be of interest to researchers and funders, and will hopefully help to guide future themed calls to promote research in these areas. The list includes questions relating to all stages of the transplant process, including access to transplantation, living donation and organ preservation, post-transplant care and management of the failing transplant. It also includes questions relating to interventions which may be further on the horizon, but are of such significance that their inclusion was deemed very important, such as the ability to bioengineer organs. Whilst the process has not necessarily identified any radical, previously unconsidered questions, it has allowed us to assign shared priorities to these unanswered questions in order to help direct future research activity and funding.

The JLA process followed has now been used to identify research priorities in over 35 areas of medicine (http://www.jla.nihr.ac.uk/), and the methodology used fulfils many of the criteria defined for good practice in priority setting [16,17]. The involvement of patients, carers and donors at all stages including the planning and design of the project ensures that the views of these groups are represented equally to those of healthcare professionals. Approximately equal proportions of professionals and non-professionals engaged in each stage of this project. Engagement of all major national professional and patient organisations and charities aided the reach of the process, and will also help with dissemination of the final list of priorities to ensure that they are addressed by future research.

The true impact of patient involvement in the PSP process is difficult as it was not formally assessed. Most of the questions included in the final workshop and top ten were submitted by a mixture of professionals or non-professionals, whilst questions submitted solely by patients were uncommon. Nonetheless, some of the questions included in the final top ten were suggested by far more patients than professionals (such as the long-term impacts of donor nephrectomy). The final top ten included questions ranked highly by all groups during the prioritisation process, with an equal mixture of those ranked highly by professionals and non-professionals. It is likely, therefore, that patient involvement played an important role in the shaping of the final prioritised list.

A recent systematic review identified four existing publications outlining research priorities with a scope including kidney transplantation [18]. Of these, one focused solely on paediatric transplantation [19], with the remainder considering all aspects of Chronic Kidney Disease (CKD) [8,20,21]. Only one of these studies involved patients and professionals in partnership, aiming to set research priorities for patients on or nearing dialysis [8]. Due to the patient population and scope, the only question relating to transplantation reaching the top ten related to improving access to transplantation. A more recent study has involved both patients and professionals in prioritising research topics for CKD patients, considering transplantation as a standalone category [9]. Potential questions were submitted by participants during a one-day workshop, with 20 questions regarding transplantation considered. Of note, there were only 9 transplant recipients included in the group of 60 participants. In contrast, the current PSP process involved over 250 participants, and considered 90 potential indicative research questions. Whilst the many of the questions considered in this previous study were also identified in the current process, the prioritisation was very different with a much greater focus on psychological health and wellbeing of donors and recipients, as well as aspects of paediatric transplantation and organ donation. This likely reflects the differences in scope, the size of the pool of questions considered, and differences in the composition and interests of the consensus groups. It is however possible that differences in healthcare settings and/or cultural differences between countries could impact the priorities identified.

The current process may under-represent certain groups affected by transplantation. In particular, very few responses were received from children and adolescents despite partnership with children’s renal charities and the British Association for Paediatric Nephrology. However, many respondents were parents of affected children or nephrologists involved in the treatment of these patients, and questions regarding child and adolescent transplantation were considered during the final workshop. The other patients under-represented in the current process are those from black, Asian and minority ethnic (BAME) groups. These groups made up around 9% of the responses to the initial survey, and 5% of those to the prioritization survey, yet represent around one quarter of the patients on the UK transplant waiting list. BAME patients have significantly higher waiting times to transplantation in the UK, and are less likely to donate [3]. Questions regarding access to transplantation in these groups were submitted to the PSP, and the indicative question “In potential kidney transplant recipients, how can we ensure fair and equal access to transplantation across the UK?” was considered in the top 25 questions during the final workshop.

A further limitation of the current process is the relatively small number of participants in the final workshop. A group size of around 25 participants was determined to provide optimal interaction based upon experience from previous JLA PSPs, and within the budget limitations of the project. Unfortunately, due to illness and unforeseen work commitments the final number of participants was reduced to 20. Despite this, the final group had fair and balanced representation from all stakeholder groups including patients, donors and clinicians. Furthermore, discussions during the final workshop were informed by the rankings of all groups during the prioritisation survey, meaning that the final decision making was influenced by a broader group than just those involved in the workshop.

Whilst the prioritised results of this PSP are most relevant to the UK healthcare setting, it is likely that the long-list of potential research questions has international relevance. The review process to check whether questions were truly unanswered by existing literature included evidence from all international healthcare settings, and so whilst it is conceivable that priorities may differ, the unanswered questions are likely to remain the same. This notion is supported by comparison of questions submitted to the current process and those submitted to previous prioritisation processes in other healthcare settings, with significant overlap seen despite differences in priority [9]. One caveat to this is that both the current and other existing prioritisation exercises were undertaken in high-income countries with well-developed and funded healthcare systems. Research priorities in developing countries may differ from those identified here.

Priority setting should not be seen as a one-time exercise, and the priorities set in this process will require future re-evaluation to ensure continuing relevant or to identify new areas of important uncertainty [16]. As yet, the JLA do not have a defined process for the re-evaluation of research priorities, and this will become more important as time passes and more healthcare areas are evaluated. The other area of paucity in the literature is the formal assessment of the impact of such priority setting exercises on the research performed, and in particular the impact of patient involvement [22]. Future evaluation of the questions addressed by successfully funded projects in the field will be essential to assess the impact of this project, although measureable impact is likely to take a number of years.

The Kidney Transplant PSP has provided an invaluable resource of prioritized, unanswered research questions to help in the design of future research studies. By increasing awareness of these topics, we hope to divert research effort and funding towards the design and implementation of high quality studies, including collaborative randomised controlled trials, with the goal of improving access to transplantation and the long-term outcomes for both donors and recipients.

## Supporting Information

S1 File List of partner organisations to the Kidney Transplant PSP. (DOCX)

S2 File Strategy for identifying systematic reviews in solid organ transplantation. (DOCX)

S3 File Full list of indicative research questions submitted to the Kidney Transplant PSP. (DOCX)

## Abbreviations

BAME: Black, Asian and Minority Ethnic
BKPA: British Kidney Patient Association
BRS: British Renal Society
BTS: British Transplantation Society
CET: Centre for Evidence in Transplantation
CKD: Chronic Kidney Disease
JLA: James Lind Alliance
KRUK: Kidney Research UK
NETSCC: NIHR Evaluation, Trials and Studies Coordinating Centre
NIHR: National Institute for Health Research
NKF: National Kidney Federation
PSP: Priority Setting Partnership
RA: Renal Association
RCT: Randomised Controlled Trial
SDC: Supplementary Digital Content
