# Supplementary material for: Defining Priorities for Future Research: Results of the UK Kidney Transplant Priority Setting Partnership
Source: PLoS One. 2016 Oct 24;11(10):e0162136. doi: 10.1371/journal.pone.0162136 (PMC5077146; doi:10.1371/journal.pone.0162136)
Supplement: S1 File — (DOCX) [file pone.0162136.s001.docx]

**S1 File. List of partner organisations to the Kidney Transplant PSP.**

Core partners (represented in the steering group)

- Centre for Evidence in Transplantation
- James Lind Alliance
- Renal Association
- British Transplantation Society
- National Kidney Federation
- British Kidney Patient Association
- Kidney Research UK

Other partners (involved in publicity and dissemination)

- Scottish Kidney Federation
- Polycystic Kidney Disease (PKD) Charity
- Carrel Club
- British Association for Paediatric Nephrology
- Give a Kidney Charity
- South Thames Kidney Fund
- South West Thames Institute for Renal Research
- Oxford Transplant Foundation
- Kids Kidney Research Charity
- Alport UK
- British Society for Histocompatibility and Immunogenetics
- UK Renal Pharmacy Group
- Efficacy and Safety of Prescribing in Transplantation (ESPRIT)
- The UK Renal Registry
