# Supplementary material for: Defining Priorities for Future Research: Results of the UK Kidney Transplant Priority Setting Partnership
Source: PLoS One. 2016 Oct 24;11(10):e0162136. doi: 10.1371/journal.pone.0162136 (PMC5077146; doi:10.1371/journal.pone.0162136)
Supplement: S2 File — (DOCX) [file pone.0162136.s002.docx]

**S2 File. Strategy for identifying existing systematic reviews.**

The Centre for Evidence in Transplantation maintains the Transplant Library database ([www.transplantlibrary.com)](http://www.transplantlibrary.com)). This resource contains randomised controlled trials, systematic reviews and clinical practice guidelines published in the field of solid organ transplantation. The Transplant Library database was used to identify published systematic reviews relevant to the research questions submitted to the Kidney Transplant Priorities Setting Partnership. In addition, the PROSPERO database (<http://www.crd.york.ac.uk/PROSPERO/)> and Cochrane Library (<http://www.cochranelibrary.com/)> were searched for registered protocols of ongoing systematic reviews.

**Search strategy for identifying systematic reviews in solid organ transplantation**

In order to maintain the Transplant Library database, comprehensive searches are performed within OVID MEDLINE and the Cochrane Library for both randomised controlled trials and systematic reviews in solid organ transplantation. Searches use a combination of subject headings and free text terms. A sample search strategy for systematic reviews in OVID MEDLINE is shown in the table below:

| **#** | **Search term(s)** |
| --- | --- |
| 1 | Organ Transplantation/ |
| 2 | exp Heart Transplantation/ |
| 3 | Kidney Transplantation/ |
| 4 | Liver Transplantation/ |
| 5 | exp Lung Transplantation/ |
| 6 | Pancreas Transplantation/ |
| 7 | or/1-6 |
| 8 | ((organ$1 or multiorgan or multi-organ or multivisceral or multi-visceral or heart$1 or cardiac or lung$1 or hepatic or liver$1 or kidney$1 or renal or pancreas or kidney-pancreas or pancreas-kidney or heart-lung or lung-heart or stomach or intestin$ or bowel$1 or colon or gastrointestinal or gut$1 or duodenum or jejun$ or ileum) adj2 (transplant$ or allograft$1 or graft$1 or recipient$1 or donor$1)).ti,ab. |
| 9 | (chronic adj allograft adj nephropathy).ti,ab. |
| 10 | (cardiac adj allograft adj vasculopathy).ti,ab. |
| 11 | (donor adj nephrectomy).ti,ab. |
| 12 | or/8-11 |
| 13 | ((living or live or cadaveric or cadaver or dead or died or non-heart-beating or (non adj heart adj beating) or heart-beating or (heart adj beating)) adj2 donor$).ti,ab. |
| 14 | ((transplant$1 or graft$1 or allograft$1) adj2 (survival or rejection)).ti,ab. |
| 15 | ((acute or chronic) adj rejection).ti,ab. |
| 16 | (transplant adj recipient$1).ti,ab. |
| 17 | Transplantation/ |
| 18 | Transplants/ |
| 19 | Immunosuppression/ |
| 20 | Graft Enhancement, Immunologic/ |
| 21 | Histocompatibility Testing/ |
| 22 | "Blood Grouping and Crossmatching"/ |
| 23 | Transplantation Immunology/ |
| 24 | Histocompatibility/ |
| 25 | exp Host vs Graft Reaction/ |
| 26 | Tissue Donors/ |
| 27 | Living donors/ |
| 28 | Organ Preservation Solutions/ |
| 29 | Organ Preservation/ |
| 30 | "Tissue and Organ Harvesting"/ |
| 31 | exp "tissue and organ procurement"/ |
| 32 | tissue preservation/ |
| 33 | Transplantation Tolerance/ |
| 34 | or/13-33 |
| 35 | (organ$1 or multiorgan or multi-organ or multivisceral or multi-visceral or heart$1 or cardiac or lung$1 or hepatic or liver$1 or kidney$1 or renal or pancreas or kidney-pancreas or pancreas-kidney or heart-lung or lung-heart or stomach or intestin$ or bowel$1 or colon or gastrointestinal or gut$1 or duodenum or jejun$ or ileum).ti,ab. |
| 36 | 34 and 35 |
| 37 | brain death/ |
| 38 | (transplant$1 or donor$1).ti,ab. |
| 39 | 37 and 38 |
| 40 | nephrectomy/ |
| 41 | transplant$1.ti,ab. |
| 42 | 40 and 41 |
| 43 | Intestines/tr [Transplantation] |
| 44 | Intestine, Large/tr [Transplantation] |
| 45 | Cecum/tr [Transplantation] |
| 46 | Colon/tr [Transplantation] |
| 47 | Rectum/tr [Transplantation] |
| 48 | Intestine, Small/tr [Transplantation] |
| 49 | Duodenum/tr [Transplantation] |
| 50 | Ileum/tr [Transplantation] |
| 51 | Jejunum/tr [Transplantation] |
| 52 | or/43-51 |
| 53 | 43 or 44 or 45 or 46 or 47 or 48 or 49 or 50 or 51 |
| 54 | 7 or 12 or 34 or 36 or 39 or 42 or 52 |
| 55 | cochrane database of systematic reviews.jn. |
| 56 | search.tw. |
| 57 | meta-analysis.pt. |
| 58 | Medline.tw. |
| 59 | Systematic review.tw. |
| 60 | or/55-59 |
| 61 | 54 and 60 |

Records identified in these searches are then screened for eligibility. Eligible references are categorised by study type and organ type, and quality assessed.

**Hand searches**

Hand searches are also performed within conference proceedings from the major transplant congresses for inclusion in the database.

**Identification of reviews relevant to PSP questions**

In order to identify existing systematic reviews relevant to the questions submitted to the Kidney Transplant PSP, an individual search strategy was developed for each question using a combination of subject headings and free text terms. These terms were then used to search the Transplant Library Database, combined with filters for systematic reviews and kidney transplantation.

For example, for the question:

“In kidney transplant recipients, which strategies prevent the formation of donor specific antibodies?”

The search strategy used was:

exp Antibodies/ OR “Donor Specific Antibod$”.tw. OR “DSA”.tw.

For the question:

“In kidney transplant recipients, does the use of ureteric stents improve outcomes compared to no stent?”

The search strategy used was:

exp Stents/ OR stent$.tw.

This process was repeated for each of the 97 indicative questions identified in the PSP.
