# Supplementary material for: Defining Priorities for Future Research: Results of the UK Kidney Transplant Priority Setting Partnership
Source: PLoS One. 2016 Oct 24;11(10):e0162136. doi: 10.1371/journal.pone.0162136 (PMC5077146; doi:10.1371/journal.pone.0162136)
Supplement: S3 File — (DOCX) [file pone.0162136.s003.docx]

**S3 File. Full list of indicative research questions submitted to the Kidney Transplant PSP.**

Recipient assessment

- In potential kidney transplant candidates, what is the best method for assessing and modifying cardiovascular risk? Does this differ for diabetic/non-diabetic patients?
- In potential kidney transplant candidates, what are the optimal investigations required to assess suitability?
- In patients on the kidney transplant waiting list, which investigations are required for ongoing assessment, and how frequently?
- In potential kidney transplant recipients, what are the absolute exclusion criteria (e.g. age, body mass index, history of malignancy, co-morbidities)?
- In kidney transplant candidates on the waiting list, what investigations are required on admission for transplant?

Recipient education

- In potential kidney transplant recipients, what is the best method for delivering pre-transplant education?

Access to the waiting list and organ allocation

- In potential kidney transplant recipients, how can we ensure fair and equal access to transplantation across the UK?
- In kidney transplantation, what is the optimum timing for a pre-emptive transplant?
- In potential kidney transplant candidates, what is the best method of deceased donor kidney allocation to ensure fair access to all age groups whilst minimising waiting times and cold ischaemia
- In deceased donor kidney transplantation, is there a robust way in which we can assess the suitability of individual organs for transplantation and predict outcomes (including DCD/ECD kidneys)?
- In deceased donor kidney transplantation, how can we ensure that organs are effectively matched to recipients (e.g. age, nephron dosing)?
- In patients on the kidney transplant waiting list, does increased patient choice (as to the organs they will accept) improve outcomes and/or access?
- In deceased donor kidney transplant recipients, in what circumstances does the recipient benefit from dual as compared to single kidney transplant?

Highly sensitised recipients

- In highly sensitised patients on the kidney transplant transplant waiting list, is it possible to desensitise patients to improve the chances of transplantation?
- In highly sensitised patients on the kidney transplant waiting list, how can we improve transplant rates?
- In highly sensitised kidney transplant recipients, how can we improve outcomes?

Organ preservation/reconditioning

- In kidney transplant recipients, what techniques to preserve, transport or recondition the kidney can lead to increased preservation times and/or improved outcomes? (e.g. normothermic/hypothermic machine perfusion, normothermic reconditioning, oxygenation)
- In kidney transplant recipients, can the addition of substances to the storage solution improve preservation and outcomes? (e.g. oxygenation, EPO, complement inhibitors, stem cells, scavenger molecules)?

Live donor transplantation

- In the assessment of potential living donors, how can we speed up the assessment process to ensure more timely transplant?
- For potential living donors, what should be the absolute exclusion criteria for donation (e.g. age, BMI)?
- In potential living donors, what are the necessary investigations to ensure safe donation whilst minimising the assessment time?
- When there is more than one potential living donor for an individual, what criteria should be used for selecting the donor to ensure optimum outcome for both donor and recipient?
- In living donor transplantation, which interventions increase the number of potential donors coming forward, and the proportion proceeding to donation?
- In live kidney donors, does laparoscopic donor nephrectomy improve outcomes compared to open or mini-incision nephrectomy?
- Following living kidney donation, what interventions reduce the time for recovery (e.g.methods of pain relief)?
- Following living kidney donation, what are the long-term health risks to the donor?
- Following living kidney donation, are there any long-term psychological consequences to the donor?
- Following living donor kidney transplantation, should donors avoid certain medications (e.g. NSAIDS)?
- Following living kidney donation, are the outcomes from pregnancy the same as in non-donors?
- Following living kidney donation, what is the best model for donor follow-up?
- In living donors entering the paired exchange scheme, how can we speed up the donation process?
- In living kidney donation, would entering all donor-recipient pairs into at least one round of the paired exchange scheme increase donation rates and improve matching?

Non-directed donation

- Do the medical and psychological impacts in non-directed living donors differ from those in directed donors?
- What interventions could increase the number of non-directed donors putting themselves forwards and proceeding to donation?
- What is the social impact of non-directed donation outside of the donor, recipients and their immediate families?
- What is the most effective way to allocate non-directly donated kidneys to maximise outcomes?
- Would reimbursement of non-directed living kidney donors increase donation rates without leading to exploitation?

Tissue typing/immunology

- In deceased donor kidney transplant recipients, what effect would more specific tissue typing have on waiting times and outcomes? (e.g. HLA-DQA, DPA and DBP matching)
- In kidney transplant recipients, how can we better assess the likely immunological response to a particular organ?

ABO/HLA incompatible transplantation

- In potential recipients of blood group (ABO) incompatible kidney transplants, which interventions reduce antibody titres and improve the safety and results of transplantation?
- In potential recipients of HLA incompatible kidney transplants, what level of HLA antibody is safe to transplant across? What factors or characteristics affect this level?

Donor specific antibodies

- In kidney transplant recipients, which strategies prevent the formation of donor specific antibodies?
- In kidney transplant recipients, does routine screening for and treatment of donor-specific antibodies improve outcomes? What is the most effective intervention?

Surgical technique/intraoperative management

- In kidney transplant recipients, does laparoscopic implantation improve outcomes compared to open implantation?
- In women with at least one normal delivery, does transvaginal kidney transplantation provide equivalent outcomes to open surgery?
- In kidney transplant recipients, do donor or reperfusion biopsies aid organ selection and/or clinical management to improve outcomes when compared to no biopsies?
- In kidney transplant recipients, what interventions at the time of transplantation improve graft outcomes?
- In kidney transplant recipients, does the use of ureteric stents improve outcomes compared to no stent?
- In kidney transplant recipients, what is the most effective method of analgesia for the postoperative period?
- In kidney transplant recipients, what is the best regimen for intravenous fluid replacement? Does this vary by transplant type or recipient characteristics?
- In kidney transplant recipients, does implantation into a structurally or functionally abnormal urinary tract affect outcomes compared to a normal urinary tract?
- In kidney transplant recipients, how can the risks of future elective surgery be minimised?

Cardiovascular/metabolic complications of transplantation

- In kidney transplant recipients, which cardiovascular medications improve outcomes (e.g. aspirin, angiotensin converting enzyme inhibitors, angiotensin receptor blockers, statins)?
- In kidney transplant recipients, what is the optimal blood pressure target? Is this the same as the non-transplant population?
- In kidney transplant recipients, can we predict those at high risk of new-onset diabetes? What interventions can minimise this risk?
- In kidney transplant recipients, how can we diagnose, prevent and/or manage renal bone disease?

Infective complications of transplantation

- In kidney transplant recipients, does antibiotic prophylaxis reduce the risk of urinary tract infection compared to no prophylaxis?
- In kidney transplant recipients, is screening for BK virus (with appropriate intervention) clinically- and cost-effective compared to no screening? What is the most effective intervention?
- In kidney transplant recipients, what is the optimum duration of CMV prophylaxis post-transplant?

Post-transplant malignancy

- In kidney transplant recipients, is screening for common malignancies clinically- and cost-effective (e.g. skin, prostate, colon, breast, cervix, post-transplant lymphoproliferative disorder)?

Acute rejection

- In kidney transplant recipients, what is the best way to manage vascular or antibody mediated acute rejection?
- In kidney transplant recipients with subclinical acute rejection, does active management improve outcomes compared to watchful waiting? Which intervention is most effective?
- In kidney transplant recipients with acute rejection, are there biomarkers that can allow early diagnosis?
- In kidney transplant recipients, what is the best way to manage acute cellular rejection?
- In kidney transplant recipients, does blood transfusion increase the risk of acute rejection?

Chronic rejection/CAN/late graft loss

- In kidney transplant recipients, what is the best strategy to prolong the life of the transplant kidney?

Delayed graft function

- In kidney transplant recipients, is there a suitable biomarker for identifying or predicting delayed graft function?

Recurrent disease

- In kidney transplant recipients, how do we prevent recurrent disease (e.g.glomeulonephritis) post-transplant?

Quality of life/psychosocial outcomes

- In kidney transplant recipients, does access to psychological or psychosocial support improve outcomes?
- In kidney transplant recipients with depression or anxiety, what is the best method for management?
- In kidney transplant recipients, what is the best method for measuring quality of life?
- In kidney transplant recipients, does recipient ethnicity affect quality of life following transplantation?

Post-transplant monitoring

- In kidney transplant recipients, do routine biopsies of the transplant kidney improve outcomes compared to on-demand biopsies?
- In kidney transplant recipients, what is the optimum follow-up protocol to minimise clinic visits whilst ensuring that problems are detected early?

Management of the failing transplant

- In kidney transplant recipients with a failing transplant, when is the optimum time to re-list on the transplant waiting list?
- In kidney transplant recipients with a failing transplant, what is the safest management strategy that minimises sensitisation - graft nephrectomy, immunosuppression withdrawal or immunosuppression continuation?

Diet and lifestyle

- In kidney transplant recipients, what lifesyle factors affect outcomes (e.g. diet, exercise, alcohol, environmental hazards, weight, medications, work)?
- In female kidney transplant recipients of child bearing age, what are the risks of pregnancy when compared to pregnancy in a non-transplanted individual? How long post-transplant is pregnancy safe?

Immunosuppression and tolerance

- In kidney transplant recipients, what is the most effective immunosuppressive regimen (e.g. azatiohprine vs mycophenolate, belatacept, generic vs proprietary)?
- In kidney transplant recipients, are generic immunosuppressive drugs as effective as proprietary (brand-name) drugs?
- In kidney transplant recipients, does belatacept improve outcomes and transplant histology in kidney transplant recipients compared to mTOR inhibitor or tacrolimus-based drug combinations?
- In kidney transplant recipients, which immunosuppressive regimens can minimise side effects (e.g. infections, diarrhoea, malignancy)?
- In kidney transplant recipients, how can we induce tolerance to the graft to prevent or reduce the need for immunosuppression (e.g. T-regulatory cells, induction of haemoxygenase 1)?
- In kidney transplant recipients, how can immunosuppression be personalised to the individual patient for optimal outcomes?
- In kidney transplant recipients, can we improve monitoring of the level of immunosuppression (e.g. T-cell or B-cell ELISPOT, point-of-care tacrolimus monitoring, MMF monitoring)?
- In kidney transplant recipients, what interventions improve compliance with immunosuppression?

Paediatric and adolescent transplantation

- In adolescent/young adult kidney transplant recipients, what interventions improve outcomes?
- In paediatric kidney transplant recipients, what are the most effective interventions to prevent sensitisation?

Artificial and bioengineered organs

- In potential kidney transplant recipients, can xenotransplantation become as safe and effective as human to human transplantation?
- In potential kidney transplant recipients, can bioengineered organs be developed to provide equivalent safety and efficacy as human to human transplants?
